# Quantitative Assessment of Primary Colonizer Adhesion on Different Resin-Based Restorative Materials Using SYBR Green qPCR

**DOI:** 10.3390/dj14070388

**Published:** 2026-06-23

**Authors:** Lea Aylin Schmitz, Kamelia Parkhoo, Stefan Heitkamp, Georgios E. Romanos, Eva Herrmann, Maria Giraki, Susanne Gerhardt-Szép

**Affiliations:** 1 Department of Operative Dentistry, Carolinum, Goethe University Frankfurt, Theodor-Stern-Kai 7, 60596 Frankfurt am Main, Germany; k.parkhoo@med.uni-frankfurt.de (K.P.); heitkamp@med.uni-frankfurt.de (S.H.); giraki@med.uni-frankfurt.de (M.G.); s.szep@em.uni-frankfurt.de (S.G.-S.); 2 Department of Oral Surgery and Implant Dentistry, Carolinum, Goethe University Frankfurt, Theodor-Stern-Kai 7, 60596 Frankfurt am Main, Germany; grperio@gmail.com; 3 Institute of Biostatistics and Mathematical Modelling, Goethe University Frankfurt, Theodor-Stern-Kai 7, 60596 Frankfurt am Main, Germany; herrmann@med.uni-frankfurt.de

**Keywords:** primary colonizers, adhesion, restorative materials

## Abstract

**Background/Objectives:** The aim of this study was to investigate primary colonizers adhering to the oral biofilm on five adhesive restorative materials. **Methods:** For each material (Admira Fusion, Clearfil AP-X, Durafill VS, Filtek Supreme XTE, and Venus Diamond), sixteen test specimens were prepared according to a standardized protocol. For pellicle formation, the specimens were incubated for two hours at 37 °C with sterile-filtered inactivated human saliva. The bacteria (*Streptococcus oralis* (*S. oralis*), *Streptococcus gordonii* (*S. gordonii*), *Streptococcus sanguinis* (*S. sanguinis*), *Streptococcus mitis* (*S. mitis*), and *Actinomyces naeslundii* (*A. naeslundii*)) were cultivated and suspended. A bacteria mix was prepared from the suspensions. The specimens with pellicles were wetted with the bacterial mix and incubated at 37 °C for 8 h. The total genomic DNA of the adhered bacteria was isolated and subsequently quantified using SYBR Green qPCR. **Results:** For *S. gordonii*, *S. oralis*, and *A. spp.*, no significant differences in the amount of adhered bacterial DNA were found between the different materials. *S. mitis* DNA concentration was significantly higher on Filtek Supreme XTE compared to the other materials. Significantly higher DNA concentrations of *S. sanguinis* could also be detected on Filtek Supreme XTE compared to Clearfil AP-AX and Durafill VS. **Conclusions:** The investigated restorative materials showed species-specific differences in bacterial adhesion, with Filtek Supreme XTE exhibiting higher adhesion of S. mitis and S. sanguinis, whereas no differences were observed for the other tested species.

## 1. Introduction

Secondary caries are one of the most common causes of composite filling failure, requiring replacement or repair [1,2,3,4]. Microbial biofilms that adhere to tooth structures or filling materials play a major role in this process and are therefore the main cause of carious lesions and gingival inflammation [5,6]. It is also known that the risk of developing secondary caries and periodontal inflammation increases with the amount of adhered plaque [7]. The extent to which biofilms adhere to restorations is therefore significant for the longevity of restorations in the oral cavity [5,8].

In biofilm formation, the adhesion of initial colonizers is an important step that also influences the later composition of mature plaque [9]. Pellicle formation is essential to the development of a pathogenic biofilm, as it enables subsequent microbial adhesion [10,11,12].

In terms of the longevity of restorations, it is important to reduce or even completely prevent bacterial adhesion [13,14,15,16]. There is growing demand for materials that minimize plaque formation [17]. In particular, in the field of dental resins, materials are used that have different surface properties due to varying ingredient compositions and particle sizes, which impacts bacterial adhesion [18,19,20]. For example, cariogenic bacterial species growth could be influenced by the monomer matrix of composites [21,22].

Previous studies on filling materials have primarily focused on *Streptococcus mutans* adherence, as it is considered the principal bacterium associated with carious plaque [23,24,25,26,27,28,29,30,31]. However, there are already studies that examine primary colonizer adherence [10,18,19,27,32,33,34,35,36,37,38,39,40]. In these studies, the bacteria are examined in mixtures [10,18,19,35,36,38,39,40] or as individual species [24,25,26,27,28,29,30,32,33,34,37,41], on surfaces coated with [10,18,19,24,25,27,29,32,33,35,36,38,39] and without [26,28,30,34,36,37,41] pellicles. The detection methods used in these studies are usually microscopic [24,25,27,32,33,34,35,36,37,41]. Quantitative investigations using polymerase chain reaction (qPCR) [10,18,42,43,44,45,46,47] are comparatively less frequent, although it was pointed out that molecular biological investigations such as PCR, real-time PCR, and DNA sequencing should be used when investigating the dental microbiome [48]. Using qPCR allows for more species-specific quantification even within a bacterial mixture where species might have a low biomass [49].

To the best of our knowledge, studies specifically addressing the adhesion of initial colonizers in a five-species mix, including streptococci and actinomycetes, to pellicle-coated adhesive filling materials are lacking. In addition, possible metabolic interactions between bacterial species during biofilm formation should be investigated [39], which underscores the need to analyze different species of initial biofilm formation as a mix. Several studies have reported using SYBR Green qPCR to detect oral bacteria and biofilms [42,43,44,45,46,47]. Nevertheless, the adhesion of early colonizers in a defined five-species model, including streptococci and actinomycetes, on pellicle-coated adhesive filling materials has not yet been sufficiently investigated. In the oral cavity, microorganisms are never isolated but occur as complex biofilms with numerous interactions. Therefore, this study has opted for a species mixture in order to mimic a more realistic situation compared to observing isolated species [50].

Thus, the aim of this in vitro study is to investigate the adhesion capacity of the primary colonizers of oral biofilm on composites of different material groups to re-evaluate the respective materials in terms of their suitability for dental restorations by comparing initial plaque affinities.

## 2. Materials and Methods

### 2.1. Test Specimen Preparation

Based on a preliminary test with all five species on ten test specimens of Filtek Supreme XTE material and case number planning, it was decided to produce *n* = 16 test specimens per material. A sample size calculation was performed prior to the study using an ANOVA F-test design with five groups. Assuming a significance level of α = 0.05, a power of 80% (β = 0.20), a common standard deviation of 1.5, and a minimum relevant difference of 2.0, the required sample size was calculated as *n* = 16 specimens per material group (total *n* = 80). All specimens were processed under identical experimental conditions within the same experimental series.

Five different adhesive filling materials were selected to prepare the test specimens, including four composites of different material classes and one ormocer (Table 1 and Table 2). The test specimens were prepared and polished according to a defined protocol, which is described in Table 1.

The adhesive materials were first heated to 50 to 60 °C in a heating cabinet (P30, Memmert GmbH, Schwalbach, Germany). Meanwhile, all surfaces and instruments that would come into contact with the materials during the test specimen preparation were disinfected, and the room was darkened for working with light-curing materials.

To form 16 test disks per material with a diameter of 10 ± 0.1 mm and a thickness of 1 ± 0.1 mm, the materials were placed in a PTFE template with a length of 140 mm, a width of 330 mm, and a height of 10 mm, which contained punches in the corresponding size. A new PTFE template was used for each material. Modeling instruments such as Heidemann spatulas and ball pluggers were used for insertion. The respective material was inserted into the mold using a compule gun and then shaped with ball pluggers.

During insertion, it was important to ensure that the material was flush with the metal splint so that there were no over- or under-contours. To achieve this, excess material at the edge was carefully scraped off.

The top of the metal splint was then placed, and the material was pressed for one minute at 2000 to 3000 bar in a hydraulic press. After removal from the press, the metal construction was opened again, and the material was polymerized with a Bluephase polymerization lamp (Bluephase G4, Ivoclar, Ellwangen, Germany) at the full intensity of >1000 mW/cm^2^ for 40 s per side. The performance of the polymerization lamp was checked regularly in a ten-test specimen cycle. Finally, the test specimens were removed from the template, and excess material was removed with a sharp scalpel.

Al_2_O_3_-coated Super-Snap polishing disks (Shofu Dental GmbH, Ratlingen, Germany) with grain sizes of 20 µm (green) and 7 µm (pink) were used for the two-step polishing of the test specimen surface. The test specimens were moistened with sterile water and polished with a contact pressure of 0.3 to 0.7 N at 10,000 rpm for one minute each, first with the green and then with the pink polishing disk. A new polishing disk was used for each test specimen. After polishing, the test specimens were first disinfected with ethanol and then immersed in 70% ethanol for one minute. After five minutes of air drying, the test specimens were stored in well plates in distilled water at 37 °C in a heating cabinet (P30, Memmert GmbH, Schwalbach, Germany) for 7 days until the start of the test.

The test specimens were produced and stored in accordance with ISO 3990 [60].

### 2.2. Preparation of Bacterial Mix

For cultivation, the bacteria stored at −80 °C in cryotubes (Table 3 and Table 4) were removed from the freezer and thawed for approximately 2 min at 37 °C in a water bath (MPC E, Mechatronik GmbH, Darmstadt, Germany) with swirling movements.

A total of 40 to 50 µL of the bacterial suspension was applied to a blood agar plate (Columbia Agar + 5% sheep blood, Biomérieux GmbH, Nürtingen, Germany) that had been brought to room temperature, spread with a sterile inoculating loop, and left to dry for 2 min. For incubation, the inoculated agar plates were stacked upside down in an anaerobic pot, which had previously been provided with an Anaerocult C packet (Merck KGaA, Darmstadt, Germany) moistened with 6 mL of distilled water. The agar plates were incubated at 37 °C for 48 h in a heating cabinet (P30, Memmert GmbH, Schwalbach, Germany). The cultivation and storage of the bacterial species complied with ISO 3990.

After 48 h, 1 to 2 colonies per species were transferred from the agar plates to a Falcon tube containing 8 mL of reduced anaerobic growth medium. This was cultivated for a further 24 h in a heating cabinet (P30, Memmert GmbH, Schwalbach, Germany).

Of the five cultivated species, 1:6 dilutions were prepared from 2 mL of bacterial suspension and 10 mL of anaerobic growth medium in 15 mL Falcon tubes. These were incubated with loose lids for 2 h in a large anaerobic chamber with two packets of Anaerocult-C (Merck KGaA, Darmstadt, Germany) at 37 °C in a heating cabinet (P30, Memmert GmbH, Schwalbach, Germany). The 1:6 dilution corresponded to an optical density of 0.2 to 0.3 at a wavelength of 600 nm, which was verified using a spectrophotometer (BioMate3S, Thermo Fisher Scientific, Waltham, MA, USA). Prior to mixing, bacterial suspensions were adjusted individually to the same optical density. To prepare the bacterial mixture, equal volumes of 7 mL of each species was added to a Falcon tube to generate the multispecies inoculum. However, it should be noted that OD600-based standardization does not necessarily correspond to identical cell numbers across different bacterial species due to species-specific differences in cell size, morphology, and optical properties. Therefore, the resulting inoculum should be considered approximately standardized rather than strictly equi-cellular.

### 2.3. Pellicle Formation on Test Specimens

The saliva required for pellicle formation was collected from five healthy subjects. The local Ethics Committee of the Medical Faculty of Goethe University Frankfurt reviewed the study protocol and confirmed that formal ethical approval was not required, as the study involved collecting unstimulated saliva from healthy adult volunteers for in vitro experiments only, without any medical intervention or risk beyond minimal discomfort associated with saliva donation. All samples were collected after obtaining informed consent from the participants and were subsequently anonymized prior to analysis. No identifiable personal data were recorded. The procedures were considered minimal risk and in accordance with applicable ethical standards. The human saliva was collected, prepared, and sterilized according to Rüttermann et al.’s protocol [65], which represents a standardized in vitro model.

The test specimens, which had been stored in water up to this point, were air-dried and transferred to new 24-well plates (one plate per material). In total, 250 µL of the inactivated saliva was pipetted onto each test specimen. The test specimens were incubated at 37 °C in a heating cabinet (P30, Memmert GmbH, Schwalbach, Germany) for 2 h to allow for pellicle formation.

### 2.4. Incubation of Test Specimens with Bacterial Mix

The liquid components of the saliva were washed off the test specimens by rinsing twice with Dulbecco’s phosphate-buffered saline (DPBS) (Gibco Thermo Fisher Scientific, Darmstadt, Germany) and pipetting, leaving only the pellicle. A total of 350 µL of the pooled bacterial mix was applied to each test specimen. The well plates with the test specimens were incubated in an anaerobic pot with 2 packets of Anaerocult-C (Merck KGaA, Darmstadt, Germany) for 8 h at 37 °C in a heating cabinet (P30, Memmert GmbH, Schwalbach, Germany).

### 2.5. DNA Isolation

After incubation, the supernatant was first removed, and each test specimen was carefully washed with 300 µL DPBS at 37 °C to remove all non-adherent bacteria. The prepared test specimens were then transferred with the adhering bacteria to new 24-well plates for DNA preparation.

DNA isolation was performed using the DNA Preparation Kit from Jena Bioscience (#PP-214-L, Jena Bioscience, Jena, Germany).

To this end, 300 µL of resuspension buffer was first added to each test specimen, followed by 2 µL of lysozyme, and the resulting solution was thoroughly mixed by pipetting up and down. This was followed by further incubation at 37 °C for one hour. The mixture was then centrifuged for one minute at 10,000× *g* (G570E, Scientific Industries, Bohemia, NY, USA). The supernatant was removed and discarded. In total, 300 µL of lysis buffer and 2 µL of RNase were added to each test specimen, and this was mixed thoroughly. Then, 8 µL of protein kinase K was added, and the solution was mixed again. The test specimens were incubated for 10 min at 60 °C. The bacterial suspension was rinsed off the test specimens and transferred to a 1.5 mL reaction vessel using a pipette. Afterwards, 300 µL of binding buffer was added to each sample and the solution was mixed thoroughly. The samples were then cooled at 4 °C for 5 min and subsequently centrifuged at 10,000× *g* for another 5 min. A column was placed on a 2 mL collection tube, mixed with 100 µL activation buffer, and centrifuged at 10,000× *g* for 30 s, and the flow was discarded. The sample, i.e., the supernatant after centrifugation, was then loaded onto the column, centrifuged again at 10,000× *g* for one minute, and the flow was discarded. A total of 500 µL of washing buffer was added to each column, which was then centrifuged again for 30 s at 10,000× *g*, and the flow was discarded. This step was performed twice. After the second run, the flow was removed, and the columns were centrifuged again for one minute at 10,000× *g* to remove any remaining washing buffer and to dry the columns. The columns were then transferred to new 1.5 mL reaction vessels, and 40 to 50 µL of elution buffer was pipetted directly onto the membrane per column. After incubating for one minute at room temperature and then centrifuging for 2 min at 10,000× *g*, the samples were stored at −20 °C until SYBR Green qPCR was performed.

### 2.6. SYBR Green qPCR

A total of 9 µL master mix, consisting of 5 µL Mastermix Plus for SYBR Green (I-NO ROX RT-2N2X-03+NR, Bio & Sell GmbH, Feucht, Germany), 2 µL primer mix (1 µL forward, 1 µL reverse), and 2 µL distilled water, was added per well. The final primer design (Table 5) (MWG-Biotech GmbH, Ebersberg, Germany; metabion GmbH, Planegg, Germany; Eurogentec, Seraing, Belgium) was calculated with Primer Express Software (Applied Biosystems, Inc., Foster City, CA, USA) for *S. mitis* and taken from the literature for the other bacteria [18,66]. In total, 1 µL of the now thawed DNA was pipetted into the master mix. The plates were sealed with MicroSeal B seal foil (Bio-Rad Laboratories, Inc., Hercules, CA, USA) and then centrifuged for 30 s at 2250 rpm (Plate Fuge, Benchmark Scientific, Sayreville, NY, USA). Subsequently, duplex SYBR Green qPCR was performed for quantitative DNA detection using Pro Rad CFX96 Optic Module C1000 Touch Thermo Cycler from Bio-Rad Laboratories, Inc. (Hercules, CA, USA). Amplification specificity was assessed by melt curve analysis following qPCR. The melt curve analysis revealed a single distinct peak for all reactions, indicating specific amplification. Primer performance was additionally evaluated using standard curves and negative controls. No relevant amplification was observed in the negative controls. qPCR-derived DNA concentrations were used as a relative surrogate parameter for bacterial adhesion and biomass rather than as a direct measure of viable bacterial cell numbers. No conversion of DNA concentration into absolute bacterial cell equivalents was performed.

### 2.7. Statistical Analysis

Statistical analysis was performed using the software “BiAs. For Windows” (version 11.12 ©1989–2023 Epsilon-Verlag) in collaboration with the Institute for Biostatistics and Mathematical Modeling at Goethe University Frankfurt am Main. For the statistical analysis, a Kolmogorov–Smirnov test was first performed to check for normal distribution. Since there was no normal distribution, the analysis was then performed using the Friedmann test with Bonferroni–Holm correction for pairwise post hoc comparisons. A significance level of *p* ≤ 0.05 was used. Graphical illustrations were created using R (Version 4.5.3, R Foundation for Statistical Computing, Vienna, Austria).

### 2.8. Surface Roughness Analysis

Surface roughness measurements were performed on one representative specimen per material using a VK-X100 laser scanning microscope (KEYENCE Corporation, Osaka, Japan). The arithmetic mean roughness (Ra) was determined for each specimen. Measurements were carried out to provide a representative assessment of the surface characteristics of the investigated materials.

## 3. Results

The materials were evaluated regarding the percentage of adhered bacteria species on the different materials, as well as the median DNA concentration of the tested bacteria adhered to the materials. Using the median instead of the mean is an advantage, as it is more robust against outliers.

### 3.1. S. gordonii

**Figure 1:**
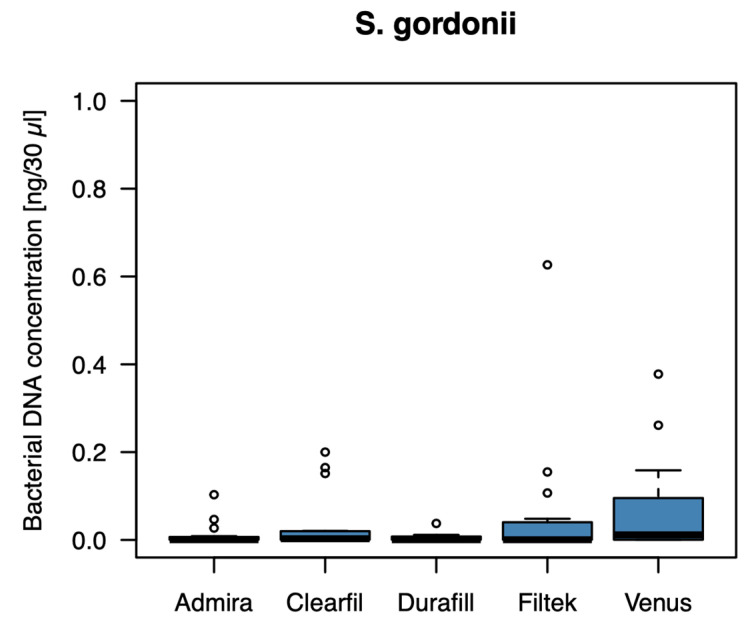
Results for *S. gordonii*. Bacterial DNA adhesion when compared between different materials. No significant differences were observed.

Different materials showed different percentages of adhered *S. gordonii* DNA on the test specimens. DNA adhered to Venus Diamond by 68.75%, to Clearfil APX by 50%, to Filtek supreme XTE by 37.5%, to Admira Fusion by 31.25%, and to Durafill by 25%. The median adhesion concentration detected on Admira Fusion, Durafill, and Filtek Supreme XTE did not differ and was, for all materials, below the limit of quantification (<LOQ). Compared to those materials, there was a higher median DNA concentration in terms of adherence to Clearfil APX (0.0069 ng/30 µL DNA) and Venus Diamond (0.0010 ng/30 µL DNA). However, there were no statistically significant differences between the materials (Figure 1).

### 3.2. S. oralis

**Figure 2:**
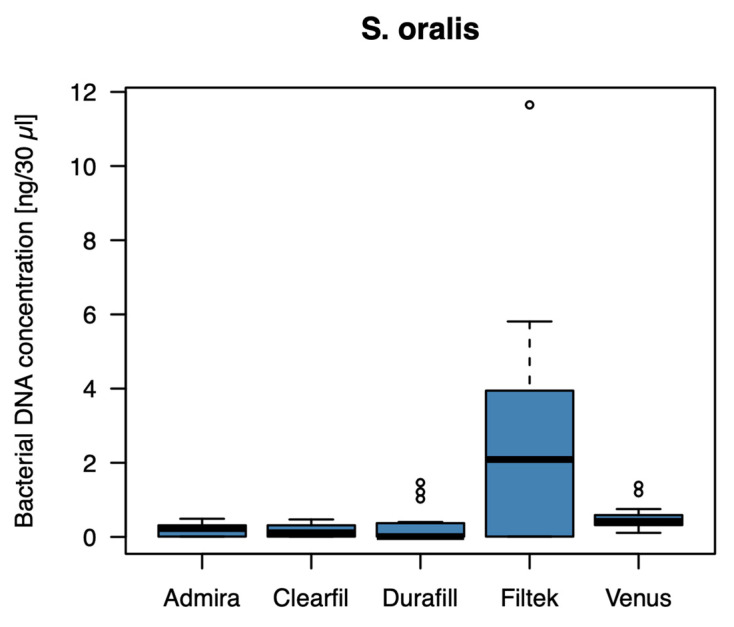
Results for *S. oralis*. Bacterial DNA adhesion when compared between different materials. No significant differences were observed.

Most of the *S. oralis* DNA adhered to Venus Diamond (81.25%), followed by Filtek supreme XTE (68.75%), Clearfil APX, Admira Fusion (62.5%), and Durafill (37.5%). The median adhesion concentration detected on Filtek Supreme XTE was 1.8138 ng/30 µL DNA, on Durafill < LOQ, and on Clearfil APX, it was 0.1222 ng/30 µL DNA. There was also no statistically significant difference compared to Admira Fusion (0.2388 ng/30 µL DNA) or Venus Diamond (0.4208 ng/30 µL DNA). A comparison between the materials revealed no statistically significant differences (Figure 2).

### 3.3. A. naeslundii-Associated Amplification

**Figure 3:**
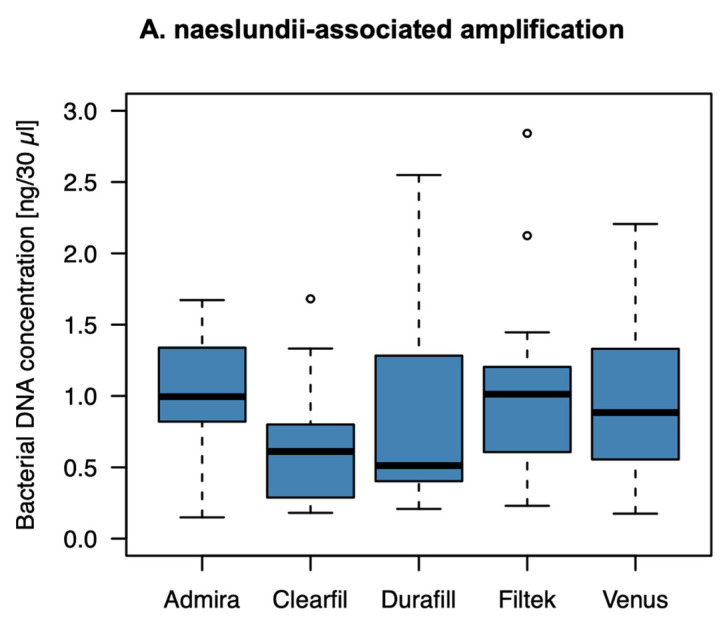
Results for *A. naeslundii-associated amplification*. Bacterial DNA adhesion when compared between different materials. No significant differences were observed.

*A. naeslundii*-associated amplification signals were detected on 100% of the test specimens for all materials. The detected median adhesion concentration differed for Admira Fusion (0.9953 ng/30 µL DNA), Clearfil APX (0.6105 ng/30 µL DNA), Durafill (0.5119 ng/30 µL DNA), Filtek Supreme XTE (1.0124 ng/30 µL DNA), and Venus Diamond (0.8839 ng/30 µL DNA), but the different results were not statistically significant when compared to each other (Figure 3).

### 3.4. S. mitis

**Figure 4:**
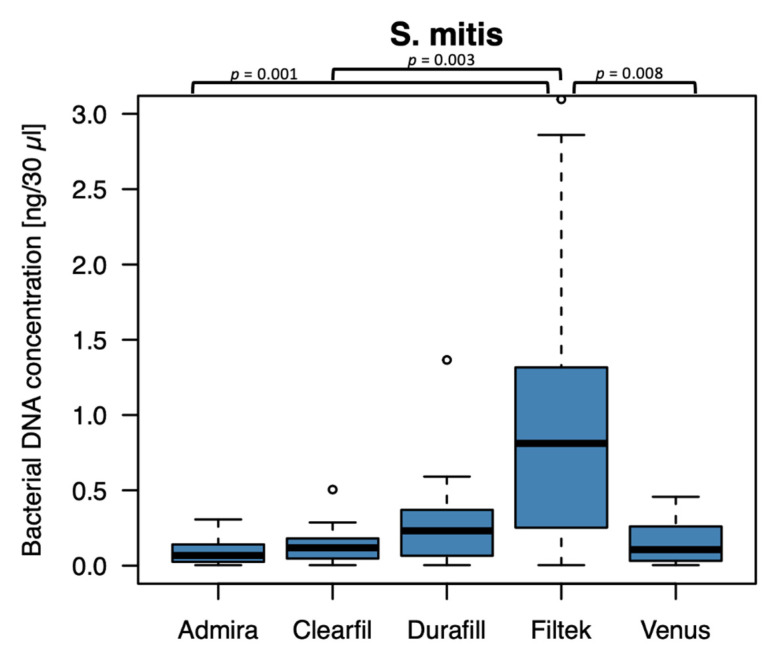
Results for *S. mitis*. Bacterial DNA adhesion when compared between different materials. Significant pairwise comparisons are marked.

*S. mitis* DNA adhered to Admira Fusion, Clearfil APX, and Filtek supreme XTE by 93.75% and on Durafil and Venus Diamond by 87.5%. Global comparison results show significant differences (*p* = 0.001). Pairwise post hoc tests showed that the detected DNA concentration was significantly higher on Filtek supreme XTE (0.8119 ng/30 µL DNA) compared to Admira Fusion (0.0681 ng/30 µL DNA; *p* = 0.001), Clearfil APX (0.1186 ng/30 µL DNA; *p* = 0.003), and Venus Diamond (0.1061 ng/30 µL DNA; *p* = 0.008). There were no statistically significant differences between Filtek supreme XTE and Durafill (0.2317 ng/30 µL DNA) or the other materials compared to each other (Figure 4).

### 3.5. S. sanguinis

**Figure 5:**
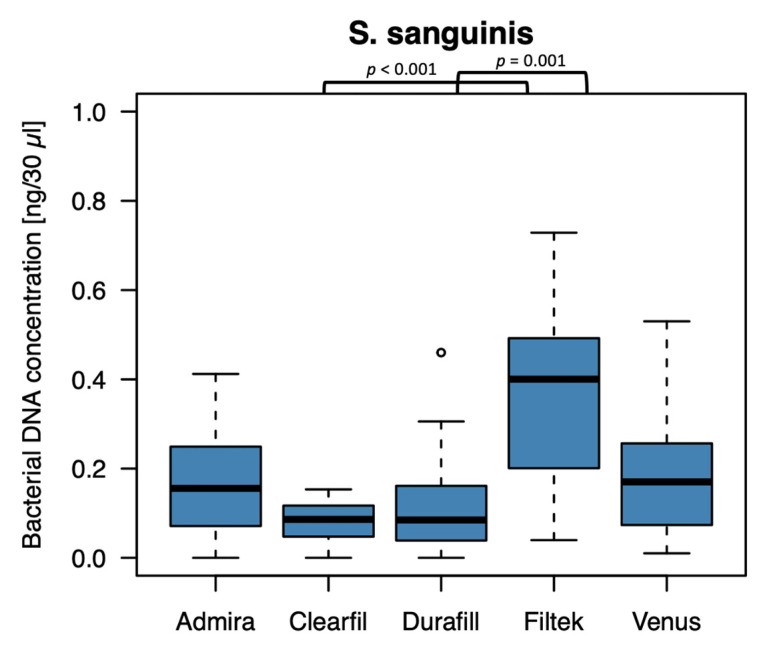
Results for *S. sanguinis*. Bacterial DNA adhesion when compared between different materials. Significant pairwise comparisons are marked.

*S. sanguinis* DNA showed high adhesion to the materials. Overall, 100% of the DNA adhered to Filtek supreme XTE and Venus Diamond and 93.75% to Admira Fusion, Clearfil APX, and Durafill. Pairwise post hoc tests showed that the detected DNA concentration was significantly higher on Filtek Supreme XTE (0.4005 ng/30 µL DNA) compared to Durafill (0.0848 ng/30 µL DNA; *p* = 0.001, *p* = 0.001) and Clearfil APX (0.0861 ng/30 µL DNA; *p* < 0.001). There was no statistically significant difference compared to Admira Fusion (0.1557 ng/30 µL DNA) or Venus Diamond (0.1702 ng/30 µL DNA). A comparison between other materials revealed no statistically significant differences either (Figure 5).

### 3.6. Surface Roughness Analysis

Considerable differences in surface roughness were observed among the investigated materials, with Filtek Supreme XTE showing the lowest roughness values and Venus Diamond exhibiting the highest. (see Table 6).

Overall, no significant differences in bacterial adhesion between the different materials are shown for *S. gordonii*, *S. oralis*, and *A. spp*. In contrast, such significant differences are observed for *S. mitis* and *S. sanguinis*. At a significance level of *p* < 0.05, there were no significant differences in DNA adhesion to the various adhesive filling materials for three of the five primary colonizers, namely for *S. gordonii*, *S. oralis*, and *A. spp*. However, the results showed a significant difference regarding *S. mitis* and *S. sanguinis* DNA adherence to different materials.

## 4. Discussion

As minimally invasive procedures in restorative dentistry are on the rise, with modern resin-based composites (RBCs) extending their field of indication and their steady improvement in esthetic and mechanical features, the question of the durability of RBCs is of increasing importance. One major aspect compromising the long-term success of RBCs is the formation of secondary caries, which may lead to restoration failure [2].

The selection of different adhesive material classes used in this study aims to represent a wide range of conventional adhesive filling materials, including ormocers, hybrid composites, microfiller composites, and nanocomposites. Due to their good mechanical and esthetic properties, these materials are the most commonly used filling materials and are therefore highly clinically relevant [67].

For this study, five primary colonizers were examined in a mixture regarding the extent of adhesion to five different filling materials. Bacteria are never isolated within the natural oral flora, and within a mixture, the adhesion of individual bacterial species can be promoted or prevented [68]. Thus, we decided to use a bacterial mix for the present study in order to mimic the natural conditions of the oral cavity and thereby provide more transferable and clinically relevant results [69]. As mentioned above, another important reason for using a mix is that primary colonizers interact with each other [70] and coaggregate, enabling and promoting adhesion to materials and biofilm formation [71]. The bacterial species selected for this study are classic primary colonizers, meaning they are among the first bacteria to colonize pellicle-coated surfaces in the oral cavity [72]. Investigating streptococci and actinomycetes in the mix is useful because these species contribute to plaque formation in different ways: streptococci stabilize the biofilm matrix, while actinomycetes contribute to its structure [73]. The species selection also represents different adhesion mechanisms, as streptococci often adhere via protein–receptor interactions, while actinomycetes can adhere to surfaces by forming a sticky polysaccharide matrix [73]. As reflected in our results where *A. naeslundii*-associated amplification signals were detected in 100% of the test specimens, previous research has shown that Actinomyces species have strong adhesion properties because of type 1 fimbriae, which allow for attachment to proline-rich proteins and type 2 fimbriae, which allow for further attachment to streptococcal structures [74]. The other streptococci species differ in their characteristics, e.g., *S. gordonii* seems to bind to amylase, an enzyme present in saliva, which may vary in amount from individual to individual and might coat different surfaces in different ways [75]. Other studies have found different adhesion capacities of different subspecies of *S. oralis*, indicating a general high variability in the adhesion capacity of bacteria, which is in accordance with our results [76]. Furthermore, the selected bacteria enable other potentially pathogenic species to colonize the biofilm [15] and can behave pathogenically themselves [77,78,79]. It has long been known that *S. mitis*, in particular, occurs in carious dentin in teeth affected by root caries [77]. On the other hand, *S. sanguinis* is associated with oral health [80] and is inversely associated with caries [81] and could have an antagonistic effect on cariogenic bacterial species such as *S. mutans* [80,81,82]. At the same time, however, *S. sanguinis* is also considered to be a cause of infectious endocarditis [78,79].

In the present study, the incubation period for the bacteria on the test specimens was selected to be eight hours. During the first six to eight hours of biofilm formation, primary colonization takes place, which lays the foundation for further biofilm growth [71]. After this time period, the maximum adhesion of primary colonizers is reached [83]. Thus, a longer incubation period of eight hours is appropriate in order to achieve and represent the maximum possible adhesion. As such, a longer incubation period is not necessary when investigating primary colonizers, as secondary colonization begins at this point [84], which exceeds the aim of our study. A shorter incubation time, on the other hand, might not fully capture the complete extent of adhesion [83].

The results revealed no statistically significant differences in bacterial adhesion between the materials for three (*S. gordonii*, *S. oralis* and *A. naeslundii*-associated amplification signals) of the five bacterial species examined. This is consistent with the results of other studies that have also investigated primary colonization and found no significant results [19,26,27,38]. Significant differences in bacterial adhesion to different adhesive materials were demonstrated in the present study for *S. mitis* and *S. sanguinis*. Regarding *S. mitis*, this contradicts the findings of Bilgili et al., who were unable to find any significant differences in *S. mitis* adhesion to the bulk-fill materials they examined [32]. Possible reasons for this could be methodological, as this study differs significantly from the present study in terms of incubation time, and *S. mitis* was examined in a monoculture rather than in a mixture [32]. In other studies that included *S. mitis* [10,18,36,39], the focus of the researchers differed from that our study, e.g., they focused on the influence of the pellicle [36] or surface roughness [34] on the interactions of different species on adhesion [39] or on investigating experimental composites [10,18]. Therefore, the results cannot be compared with those of the present study. However, in a study where adhesion on rough and smooth titanium surfaces were examined, *S. mitis* adhered significantly less to the rough surface compared to the smooth one [85]. However, Actinomyces species showed no significant difference in adhesion between the different surfaces [85]. Those results may be comparable to the findings in our study, where *S. mitis* appeared to be more adherent to the less rough nano-textured surface compared to the other materials, while no significant difference was found for *A. naeslundii*-associated amplification signals. Another study has also found *S. mitis* to adhere more to smoothened denture resin surfaces [86].

Studies on bacterial adhesion can only be compared to a limited extent due to a wide variability in methodology and a large variance in the definition of composite material groups, both in the literature and in manufacturer specifications. In addition, the results of the present study should be interpreted and considered within the limitations of an in vitro study. The results are specific to the applied in vitro conditions and should be interpreted with caution when extrapolating to the clinical in vivo situation. This study was conducted as an in vitro experiment in the laboratory under static conditions, which can only represent the conditions prevailing in the oral cavity to a limited extent. Therefore, this study’s design lacks dynamic factors such as salivary flow, pH and temperature fluctuations, chewing pressure, and abrasion or attrition processes which could influence bacterial adhesion [87,88]. In addition, this study focuses on a selection of five primary colonizers, although there is a much greater diversity of bacterial species in the intraoral environment [89]. *Fusobacterium nucleatum* should be mentioned here as an important bacterium that connects the primary colonizers and thus acts as a bridge builder [90]. It is therefore an important component of initial biofilm formation, as it is able to aggregate with many other species [90].

A further limitation is that inoculum standardization was based on OD600 adjustment and equal-volume mixing rather than direct cell quantification. Since the relationship between optical density and the actual cell number may vary between species, minor differences in the initial bacterial composition of the multispecies inoculum cannot be excluded.

An additional limitation of the present study is that no systematic physicochemical surface characterization (e.g., contact angle measurement, or surface free energy) was performed, although these parameters are known to substantially influence bacterial adhesion to restorative materials [15]. Although all specimens were polished using a standardized two-step polishing protocol under controlled pressure and rotational speed, subtle differences in filler size, filler distribution, and matrix composition may still have resulted in differences in surface topography and hydrophilicity, as variations in composite formulation have been shown to affect surface characteristics after polishing [91]. These parameters are known to influence protein adsorption and subsequent bacterial adhesion [92]. Therefore, the observed differences in adhesion, particularly for *S. mitis* and *S. sanguinis*, should be interpreted cautiously, as the underlying mechanisms cannot be determined based on the present data. It cannot be concluded whether the observed differences were primarily related to material composition itself or to associated physicochemical surface properties since they were not systematically characterized in this study.

Additional surface roughness measurements were conducted. The investigated materials exhibited pronounced differences in roughness, ranging from Ra = 0.20 µm for Filtek Supreme XTE to Ra = 1.74 µm for Venus Diamond. However, the pattern of bacterial adhesion did not correspond to the ranking of surface roughness values. Notably, Filtek Supreme XTE showed the lowest roughness while simultaneously exhibiting significant adhesion levels for *S. mitis* and *S. sanguinis*.

These findings suggest that surface roughness alone does not explain the observed species-specific adhesion behavior. Instead, material-dependent surface chemistry is likely to be a more relevant factor as the investigated materials differed substancially regarding filler morphology and matrix composition (see Table 2). Filtek Supreme XTE is characterized by a nanofilled structure composed of non-agglomerated silica nanoparticles and zirconia/silica nanoclusters (see Table 2). Since all specimens were covered with an identical salivary pellicle prior to bacterial exposure, differences in pellicle composition are unlikely to account for the observed differences in adhesion. Rather, variations in the underlying substrate properties may have influenced protein–surface interactions and thereby indirectly affected bacterial attachment.

Although marked differences in surface roughness were found among the investigated materials, these variations did not reflect the bacterial adhesion patterns observed in the present study. Future research should therefore further investigate the interplay between material composition, salivary pellicle formation, and bacterial adhesion to achieve a more comprehensive understanding of the factors governing early biofilm development on restorative materials. No surface chemical characterization was performed in this study. Techniques such as Fourier-transform infrared spectroscopy (FTIR) or energy-dispersive X-ray spectroscopy (EDX) were therefore not included, which restricts a more detailed interpretation of the observed effects. Similar future investigations should incorporate surface chemical analyses to better elucidate the mechanisms governing bacterial adhesion on restorative materials.

A limitation of the present study is that saliva processing, including pooling, filtration, heat inactivation, and dilution, may alter the composition and biological properties of the acquired pellicle. Previous studies have demonstrated that pellicle formation and composition are highly selective and sensitive to experimental conditions and saliva processing. [93,94]. Although this standardized in vitro approach improves reproducibility and comparability between experiments, it may reduce the physiological relevance of the naturally formed oral pellicle and its complex protein activity.

Additionally, there are noteworthy limitations regarding the qPCR. Substrate contamination is a major limitation for qPCR. It has been shown that even contamination within reagent kits, buffer, or water may be common [95]. Other potential sources of contamination include cross-contamination between the samples or through aerosolized amplification products from PCR that may contaminate the laboratory environment, including reagents and equipment [96]. Furthermore, internal contamination is possible, as potential untargeted primers are amplified during PCR, serving as substrates for following cycles and causing large amounts of the amplified product; this phenomenon is described as carryover [97]. Therefore, validating primers and using negative controls should be highly encouraged in order to prevent data falsification from PCR [98]. In contrast to qPCR, real-time PCR (RT-PCR) eliminates carryover risk, as signals are directly measured and scrutinized [99]. However, as our aim was to evaluate the mere DNA concentration of bacteria and not specific gene expression, qPCR was appropriate for this study. When interpreting the results, a further limitation should be considered. Although the assays were applied consistently across all experimental groups, the absence of formal validation parameters (primer efficiency, R^2^, LOD, and LOQ) limits a comprehensive assessment of assay performance.

Additionally, qPCR-derived DNA concentrations represent indirect surrogate measures of bacterial biomass and do not necessarily reflect viable bacterial cell counts. Values below the quantification threshold should therefore not be interpreted as the complete absence of bacteria. Furthermore, the quantitative determination of bacterial DNA using SYBR Green qPCR does not provide any information about the vitality of the adhering bacteria; this could be determined by further investigations such as live-dead staining.

## 5. Conclusions

A final statement on the recommended specific filling materials cannot be given. Due to the significance of secondary caries as a common cause for the need to replace composite fillings [2] and the fact that bacterial adhesion significantly increases the risk of developing secondary caries [7], further research on this topic is of great interest. In addition, with the EU-wide ban on amalgam from 2025, materials such as self-adhesive composites, self-adhesive composite hybrids, glass carbomers, alkasites, and giomers may gain importance, as they could be used as amalgam alternatives in standard care. Thus, investigating the adhesion of initial colonizers to these materials could contribute to their establishment. The investigation of other filling materials such as glass ionomer cements, other composites, or typical materials used in pediatric dentistry, such as compomers, is also useful for gaining further insights and improving the comparability of studies.

Moreover, the composition of the bacterial mix could be adjusted to the same concentration ratio as they occur in vivo. In future studies that include more complex microbial communities, secondary colonizers or bridge species like *Fusobacterium nucleatum* are needed to simulate the oral biofilm formation in a more sufficient way.

Further research to investigate the results of this study, e.g., using experimental composite formulations, is needed to improve the material. In the long term, the aim should be advancement and optimization, resulting in materials that are less susceptible to plaque.

The transfer of the in vitro study protocol to in vivo studies may also be of interest as a future research approach. Study protocols with a split-mouth design and in situ carrier splints are conceivable here.

## Figures and Tables

**Table 1:** Materials used in this investigation with material classes and batch numbers.

Product	Material Class	Batch	Company
Admira Fusion	Ormocer	2044350	Voco, Cuxhaven, Germany
Clearfil AP-X	Hybrid Composite	9E0731	Kuraray, Chiyoda, Japan
Durafill VS	Microfiller Composite	K010230	Kulzer, Hanau, Germany
Filtek Supreme XTE	Nanocomposite	NC10955	3M Espe, Landsberg am Lech, Germany
Venus Diamond	Ultra-fine particle hybrid composite	K010201	Kulzer, Hanau, Germany

**Table 2:** Characteristics of tested resin-based composites. Vol% = volume percent; wt% = weight percent; Bis-GMA = bisphenol A-glycidyl methacrylate; UDMA = urethane dimethacrylate; TEGDMA = triethylene glycol dimethacrylate; Bis-EMA = bisphenol-A-dimethacrylate-polyethylenglycole-diether; TCD-DI-HEA = bis(acryloyloxymethyl) tricyclo [5.2.1.02.6] decane; PEGDMA = polyethylene glycol dimethacrylate.

	Durafil VS	Venus Diamond	Clearfil APX	Filtek Supreme XTE	Admira Fusion
Filler size (in micrometers)	0.02–0.07 [51] prepolymer < 20 [52]	0.005–20 [53]	0.02–17 [54]	0.004–0.01/Cluster = 0.02 [55]	0.01–1 [56]
Filler content	66 vol% [51]	36.5–65.1 wt% Ba-Al-F-silicate glass, ytterbium–fluoride, silicium oxide [52,53]	86 wt% [54] Silanated barium glass, silanated colloidal silica, silinated silica [52]	78.5 wt% Fillers: non-agglomerated nanosilica and agglomerated zirconia/silica nanocluster [55]	84 wt% Silicone oxide [57]
Monomers	Bis-GMA, UDMA, TEGDMA [52]	Bis-GMA, TEGDMA [52], Bis-EMA, TCD-DI-HEA, UDMA [58]	Bis-GMA, TEGDMA [52]	Bis-GMA (5–10 wt%), UDMA (5–10 wt%), TEGDMA (5–10 wt%), Bis-EMA6 (1–10%), PEGDMA resins [59]	Resine Ormocer Silicone oxide [57]
Further ingredients	Titandioxide, fluorescent pigments, metal dioxide pigments, organic pigments, aminobenzoic acid ester, campherquinone, butylhydroxytoluene [51]	Rheology modifier, initiator system, stabilizers, pigments [53]	Dl-Campherquinone [54]		Butylhydroxytoluene [57]

**Table 3:** Species used in this investigation with strain specifications.

Species	Strain
*A. naeslundii* *A. spp.*: *A. oris*	DSM 43013 +. 1:1 DSM 23056
*S. mitis*	DSM 12643
*S. oralis*	DSM 20627
*S. sanguinis*	DSM 20068
*S. gordonii*	DSM 6777

**Table 4:** Characteristics of the tested bacteria. Ssp = streptococcal surface protein; PRP = proline-rich protein; H_2_O_2_ = hydrogen peroxide.

	*S. mitis* [61]	*S. sanguinis* [61]	*S. oralis* [61]	*S. gordonii* [61]	Actinomyes [62]
Morphology	Cocci	Cocci	Cocci	Cocci	Filamentous rods
Gram stain	Gram-positive	Gram-positive	Gram-positive	Gram-positive	Gram-positive
Oxygen requirement	Facultative anaerobe	Facultative anaerobe	Facultative anaerobe	Facultative anaerobe	Anaerobic—Facultative anaerobe
Adhesion	Pellicle	Pellicle/Alpha-amylase [63]	Pellicel	Strong adhesins (SspA/SspB) to acidic PRPs [63,64]	Fimbriae-mediated
H_2_O_2_ production	Low	High	Low	Possible	
Growth rate	Moderate–fast	Fast	Fast	Fast	Slow

**Table 5:** Design of the specific primers used in this investigation.

Species	Primer	Reference
*A. naeslundii*	F GGT CTC TGG GCC GTT ACT GA R TGG CCC CCA CAC CTA GTG	Henrich et al., 2016 [18]
*S. mitis*	GAGCTTGCTTCTCCGGATGA AATTGCACCTTTTAAGCAAATGTCA	Henrich et al., 2016 [18]
*S. oralis*	F TCC CGG TCA GCA AAC TCC AGC C R GCA ACC TTT GGA TTT GCA AC	Hoshino et al., 2004 [66]
*S. sanguinis*	F GGA TAG TGG CTC AGG GCA GCC AGT T R GAA CAG TTG CTG GAC TTG CTT GTC	Hoshino et al., 2004 [66]
*S. gordonii*	F CTA TGC GGA TGA TGC TAA TCA AGT G R GGA GTC GCT ATA ATC TTG TCA GAA A	Hoshino et al., 2004 [66]

**Table 6:** Surface roughness (Ra) of the investigated materials.

Material	Ra (µm)
Clearfil APX	1.30
Venus Diamond	1.74
Admira Fusion	0.35
Durafill VS	0.68
Filtek Supreme XTW	0.20

## Data Availability

The data presented in this study are available from the corresponding author upon request.
